# Application of CRISPR/Cas gene editing for infectious disease control in poultry

**DOI:** 10.1515/biol-2025-1095

**Published:** 2025-05-20

**Authors:** Mahdi Gallala

**Affiliations:** Animal Resources Department, Ministry of Municipality, Doha, State of Qatar

**Keywords:** gene editing, CRISPR/Cas, poultry, infectious disease prevention and control, vaccines, disease-resilience

## Abstract

The poultry industry faces multifaceted challenges, including escalating demand for poultry products, climate change impacting feed availability, emergence of novel avian pathogens, and antimicrobial resistance. Traditional disease control measures are costly and not always effective, prompting the need for complementary methods. Gene editing (GE, also called genome editing) technologies, particularly CRISPR/Cas9, offer promising solutions. This article summarizes recent advancements in utilizing CRISPR/Cas GE to enhance infectious disease control in poultry. It begins with an overview of modern GE techniques, highlighting CRISPR/Cas9’s advantages over other methods. The potential applications of CRISPR/Cas in poultry infectious disease prevention and control are explored, including the engineering of innovative vaccines, the generation of disease-resilient birds, and *in vivo* pathogen targeting. Additionally, insights are provided regarding regulatory frameworks and future perspectives in this rapidly evolving field.

## 1 Introduction

The term “poultry” encompasses a wide variety of domesticated avian species raised for eggs, meat, and feathers. These include chickens, Muscovy ducks, mallard ducks, turkeys, guinea fowl, geese, quail, pigeons, ostriches, and pheasants. Chickens dominate globally, comprising 94% of the world’s poultry population in 2020 and contributing 90 and 93% to poultry meat and egg production, respectively. Other species play regionally significant roles; for example, ducks are prevalent in Asia, turkeys are concentrated in North America and Europe, and guinea fowl and geese are primarily found in Africa and Asia [1].

Poultry are raised globally under systems ranging from simple shelters in rural areas to fully automated, large-scale operations. In developing countries, indigenous poultry often rely on foraging and minimal management, making intensive rearing economically unviable due to low productivity. In areas with limited consumption growth, such as parts of Africa, family-level production remains significant, often led by women [2]. Commercial production systems dominate globally, producing most poultry meat and eggs. These systems utilize selected breeds requiring optimal nutrition, disease prevention, and confinement. Their efficiency is driven by poultry’s high feed conversion, rapid reproduction, and short production cycles, enabling quick responses to demand and advancements in genetics, health, and feeding practices. Sophisticated housing is generally limited to large-scale operations due to cost [3].

Poultry serve as the predominant source of animal proteins [3,4], with global hen egg production reaching 86 million tonnes in 2021 [5], and over 140 million tonnes of poultry meat produced in 2023, representing 40% of the world’s total meat production [6]. However, the poultry industry grapples with multifaceted challenges, from the escalating demand for poultry products due to population growth to the dwindling availability of feed resulting from climate change and the growing allocation of arable lands for human food cultivation. Additionally, the emergence of novel avian pathogens, characterized by heightened virulence and adaptability, poses a significant threat to flock health and production [7–9]. Compounding these challenges is the alarming rise of antimicrobial resistance, presenting a field-wide problem with bacterial diseases in poultry becoming increasingly challenging to treat due to this resistance [10]. This issue is further exacerbated by the stringent regulations and evolving restrictions on the use of antimicrobials in poultry production [11–13], which have traditionally been pivotal in disease management strategies.

Traditional strategies for controlling infectious diseases in poultry have relied on a multifaceted approach encompassing biosecurity protocols [14], stringent sanitation practices, widespread vaccination initiatives [15], routine testing, and sanitary culling efforts [16]. This comprehensive framework has demonstrated varying degrees of success in managing numerous significant poultry ailments. Notably, certain diseases, such as pullorum disease and fowl typhoid, have been effectively eradicated from commercial poultry populations in several developed regions owing to the diligent implementation of these control measures [17].

Despite this effectiveness, these approaches have some drawbacks and limitations. One significant concern is their substantial cost. The expenditure can vary significantly depending on several factors, such as the scale of the operation, the specific disease being targeted, and geographic location. The financial burden can be particularly high during disease outbreaks, where rapid response efforts, increased surveillance, and mass culling may be required to prevent further spread. For instance, during outbreaks, the culling of large numbers of birds is often necessary, resulting in significant economic losses. Furthermore, this practice can also impact food security. A prime example is the High Pathogenic Avian Influenza (HPAI) epidemic in Europe, where approximately 50 million birds were culled within the span of a year, from October 2021 to September 2022, in affected farms [18].

The ongoing evolution of pathogens and the emergence of novel strains have severely undermined the efficacy of conventional methods, especially vaccination programs [19]. Consequently, there have been substantial economic losses attributed to decreased productivity, increased mortality rates, and the requirement for costly disease management measures. Compounding this issue is the lack of protective vaccines for specific diseases, further exacerbating the challenge. Lymphoid leukosis, a neoplastic disease, stands out as a prime example, as no treatments or vaccines are currently available to mitigate its impact [20].

Additionally, some poultry-related microbes pose a potential threat to human health through food safety issues, exposing the public to contaminated meat and eggs; this is the case, for example, for non-typhoidal *Salmonella* [21,22] and *Campylobacter* [21]. Additionally, other poultry pathogens, such as some avian influenza viruses, can be transmitted to humans, posing also a public health concern [23].

As a result, the poultry industry faces an urgent need for complementary control methods to bolster its defenses against infectious diseases. In this regard, genome editing (GE) techniques offer a promising avenue for innovation [24]. The Clustered Regularly Interspaced Short Palindromic Repeats/CRISPR-associated protein 9 (CRISPR/Cas9) system represents one of the gene-editing technologies that have the potential to revolutionize disease control strategies in poultry [25]. One of its primary advantages lies in its capacity to develop disease-resilient poultry by precisely modifying the host’s genome [26]. Additionally, it facilitates the engineering of innovative vaccines [27], while its ability to target pathogens *in vivo* holds considerable therapeutic promise, opening new avenues for combating poultry diseases with unprecedented precision and effectiveness [28]. Thus far, significant advancements in utilizing CRISPR/Cas technology have been achieved primarily in two poultry species, namely chicken and quail, with chicken leading the progress.

This article provides a comprehensive and up-to-date examination of CRISPR/Cas gene editing in poultry infectious disease control. It synthesizes recent research with a focus on practical applications, including the precise engineering of disease-resistant poultry, the development of next-generation vaccines, and *in vivo* pathogen targeting. CRISPR/Cas9 is highlighted as a transformative tool for advancing poultry health and improving disease management strategies.

While much of the existing work primarily addresses viral infections, this article takes a more integrative approach by extending the discussion to bacterial and protozoal diseases. By bridging multiple facets of CRISPR research in poultry, it offers a broader and more comprehensive perspective on its potential for disease control across different poultry species.

Additionally, it examines the challenges of translating this technology into commercial practice, addressing regulatory and ethical considerations, feasibility constraints, and the evolving legal landscape.

## 2 Modern gene-editing techniques

### 2.1 Overview

GE technology enables precise modifications to the genetic material, allowing for the addition, removal, or alteration of DNA or RNA within the genome [29–31].

Compared to conventional transgenic techniques, where exogenous DNA, typically recombinant DNA sequences, is randomly inserted into the genome, GE techniques offer distinct advantages. They enable the introduction of site-specific mutations without introducing additional genetic mutations into the genome, potentially yielding modifications that are indistinguishable from naturally occurring variants.

Modern GE techniques harness programmable DNA nucleases, known as genome editors, capable of inducing precise double-strand breaks (DSBs) at targeted locations within the genome, earning them the title of site-directed nucleases [32]. This capability allows researchers to precisely modify specific DNA sites, enabling a wide range of applications [33,34]. The DSBs triggered in the genome activate endogenous DNA repair mechanisms, resulting in specific genetic alterations through two main pathways: non-homologous end joining (NHEJ) [35,36] or homology-directed repair (HDR) [37]. NHEJ, the most common cellular repair mechanism, is also highly error-prone and often leads to insertions and deletions (indels) at the repair site resulting in frameshifts and functional knockouts (KO). This process can be utilized to create null mutation alleles for multiple purposes such as studying gene function. In contrast, HDR is a less frequent mechanism but it enables knock-in (KI) strategies. Depending on the objective-whether it is to insert a gene or inhibit gene function-HDR or NHEJ is preferred, respectively [38].

Site-directed nucleases encompass zinc-finger nucleases (ZFNs), transcription activator-like effector nucleases (TALENs), and CRISPR/Cas9 nucleases. Their emergence has revolutionized the realm of genetic modification in both plants and animals including poultry, primarily due to the high efficiencies achieved in targeted mutagenesis.

ZFNs, created in 1996, combine zinc finger modules with the DNA cleavage domain of the restriction enzyme FokI [39]. This fusion allows ZFNs to induce DSBs efficiently, leading to widespread use in GE across various organisms since 2001 [40–48].

The advent of TALENs in 2010 represented another pivotal advancement in the realm of designer nucleases [49,50]. Unlike ZFNs, TALENs utilized DNA-binding modules derived from TALE proteins, which offered greater flexibility and ease of generation [51].

Finally, CRISPR/Cas9 emerges as the most prevalent and advanced technique for GE. The construction of the CRISPR/Cas9 system is less expensive and intricate process compared to the ZFNs and TALENs systems, requiring mainly the synthesis of a short specific RNA sequence molecule for GE at a specific locus. Moreover, this system is more effective due to its broader accessibility to target sites and higher target specificity. This is attributed to the availability of computational tools for designing guide RNAs (sgRNAs) in the CRISPR/Cas9 system which enhances the predictability of guide specificity and contributes to minimizing off-target effects [52,53].

### 2.2 The rise and evolution of CRISPR/Cas technology

CRISPR/Cas technology has transformed the field of GE, recognized as the “Breakthrough of the Year” in 2015 by the journal *Science* [54]. The widespread adoption of the CRISPR/Cas system for GE has been propelled by its simplicity, specificity, efficiency, precision, and capability for multiplex targeting. This rapid adoption is further fueled by the open accessibility of the technology, granting researchers quick and affordable access to cutting-edge tools for their projects [55].

#### 2.2.1 Discovery and development

The CRISPR system was first identified in *Escherichia coli* in 1987 [56], with further research revealing its presence in other bacteria and archaea [57]. By 2007, it was understood as a bacterial defense mechanism against phages [58], acting similarly to an adaptive immune system [59–61]. In 2012, the components necessary for GE were identified, including CRISPR RNA (crRNA) and trans-activating crRNA (tracrRNA), which guide the Cas9 protein to specific DNA targets to introduce double-stranded breaks [62,63]. This breakthrough led to the development of the single guide RNA (sgRNA) system, simplifying the application of CRISPR/Cas9 in GE [64]. This fundamental discovery paved the way for extensive applications across various organisms, including poultry.

The discovery of CRISPR/Cas9 sparked a competitive drive to apply the system in eukaryotic cells. By January 2013, multiple research teams had demonstrated successful GE in human cells [65–68], and the first applications on germline cells soon followed [69]. That same year, the dCas9 protein, a version of Cas9 lacking nuclease activity, enabled gene regulation techniques like CRISPR activation (CRISPRa) [70] and interference (CRISPRi) [71] by fusing dCas9 with transcription regulators. In 2015, the discovery of SaCas9, a compact Cas9 variant suitable for delivery in adeno-associated viruses, expanded the system’s versatility [72]. While the Cas12a (Cpf1) protein broadened the range of targetable sites [73,74]. By 2016, base editing emerged [75] to allow precise DNA modifications without double-strand breaks, reducing off-target effects [76]. In 2017, the CRISPR/Cas13 system was identified [77], allowing RNA editing [78,79] and modifying gene function through mRNA degradation [80,81]. Further improvements led to Cas9 variants with expanded targeting flexibility and higher fidelity [82–84]. Prime editing, introduced in 2019 [85], is built upon base editing, enabling precise edits – including substitutions, insertions, and deletions – without double-strand breaks or donor templates.

**Figure 1:**
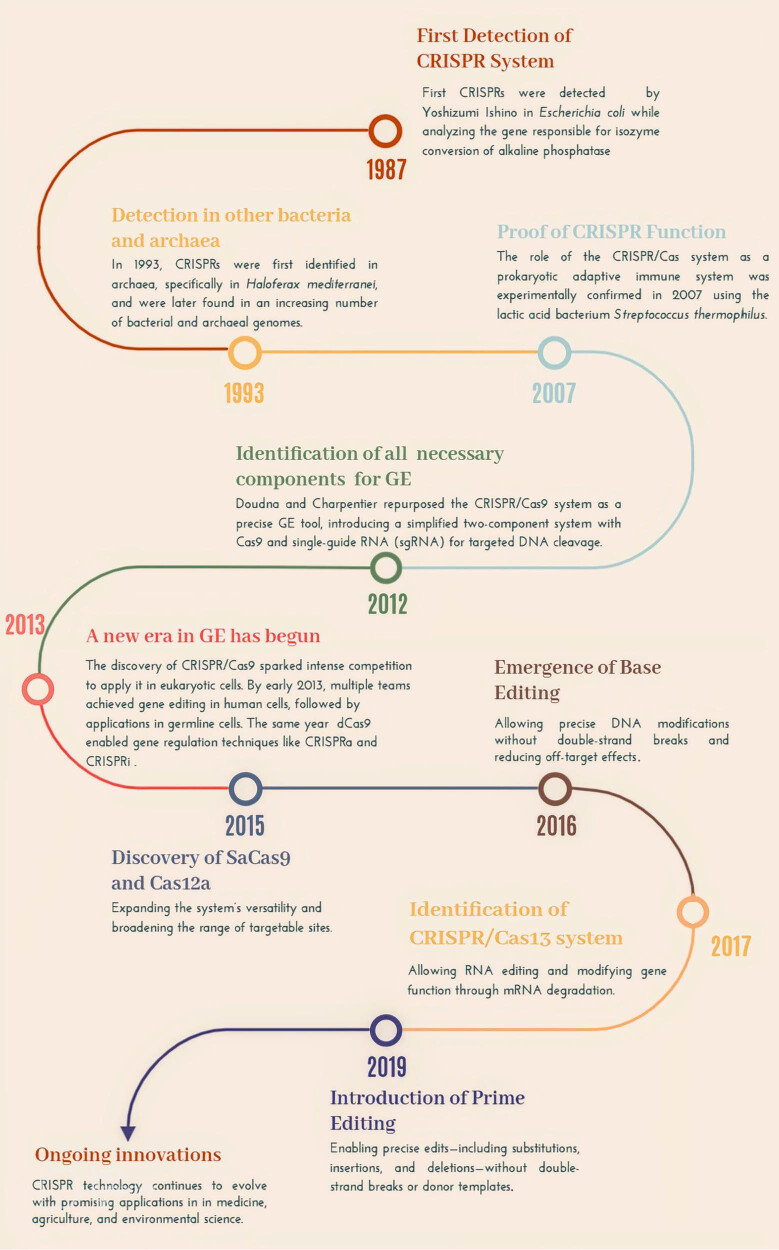
Discovery and development of the CRISPR/Cas systems.

This timeline of discoveries and advancements highlights the rapid evolution of CRISPR technology. A summary of the key milestones is provided in Figure 1.

#### 2.2.2 Classification of the CRISPR/Cas systems

In recent years, there has been a significant rise in the number and diversity of identified CRISPR/Cas systems. Based on the Cas gene signature and the specific targeting mechanism, the new classification categorizes CRISPR/Cas systems into 2 classes, 6 types and 33 subtypes [86], compared with 5 types and 16 subtypes in 2015 [87].

CRISPR/Cas9 system is the most widely used and extensively studied system for GE. This system gained prominence due to its simplicity, efficiency, and versatility in targeted GE [62].

#### 2.2.3 Limitations of the CRISPR/Cas systems

One major concern with using CRISPR/Cas9 GE technology is the potential for off-target effects, where unintended genetic modifications arise due to the system acting on sites similar but not identical to the target sequence [88]. These off-target effects occur when the Cas9 protein binds to protospacer adjacent motif (PAM)-like sequences and/or when the guide RNA (gRNA) binds to sequences that share similarity with the target site, especially when only a few base pair mismatches are present [89]. Such unintended alterations can result in small insertions and deletions (indels) or even large structural variations, such as chromosomal rearrangements. While some mutations may be biologically silent, others can have unpredictable effects, some of which may be harmful to the host, including the disruption of essential genes, immune responses, or oncogene activation [89–91].

To mitigate these risks, multiple strategies have been implemented. One approach involves utilizing bioinformatics tools to predict and assess potential off-target sites, allowing for the design of gRNAs with improved specificity [52,92]. In parallel, researchers have explored the use of Cas9 orthologs such as SaCas9 [93], St1Cas9 [94], and St3Cas9 [95], which are derived from bacterial species other than *Streptococcus pyogenes* (the source of the commonly used wild type [WT] SpCas9). These orthologs recognize more complex PAM sequences, thereby reducing the likelihood of unintended DNA cleavage. Moreover, some Cas9 orthologs exhibit inherent differences in nuclease activity and guide RNA interactions, further enhancing precision. Another refinement involves engineering high-fidelity SpCas9 variants such as eSpCas9 [83], SpCas9-HF1 [96], and HypaCas9 [97], which have been specifically designed to reduce off-target cleavage while preserving on-target efficiency. Additionally, the use of Cas9 nickases – mutant variants that introduce single-strand rather than double-strand breaks – enhances specificity. By requiring two gRNAs to target opposite strands near the desired editing site, this strategy significantly reduces off-target effects compared to conventional Cas9 [98,99].

Optimizing delivery methods also plays a crucial role in improving specificity. One effective approach is the use of Cas9-sgRNA ribonucleoproteins (RNPs), which offer rapid cellular clearance compared to plasmid-based delivery systems. Because RNPs degrade quickly after GE, they minimize the window for unintended edits, thereby enhancing specificity [100]. Similarly, the direct injection of an adenoviral CRISPR/Cas9 vector into quail blastoderm allowed precise GE as no mutations were detected in off-target regions, and vector integration was avoided [101].

Employing anti-CRISPR proteins temporarily inhibits Cas9 activity after the desired edit is made, thereby reducing the duration of potential off-target effects [102]. Moreover, given that off-target effects may vary depending on the cell cycle stage, synchronizing cells to a specific phase during CRISPR editing has been proposed as a means to further minimize errors [103].

Base editing [75,104] and prime editing [105] techniques offer alternative CRISPR methods with potentially fewer off-target effects compared to conventional Cas9-mediated DSBs.

Finally, validating CRISPR/Cas9-mediated gene editing through sequencing-based approaches [106] is critical for assessing both the intended and unintended genetic modifications. However, no single molecular assay can fully capture the genetic landscape of edited organisms or address all possible allele variations. A comprehensive molecular characterization is necessary to gain an in-depth understanding of the genetic changes induced. If undesired mutations are detected, iterative optimization of the CRISPR system can be performed to enhance precision and further reduce off-target effects [107].

## 3 Application of CRISPR/Cas gene-editing techniques in poultry

The last 15 years have witnessed the rapid development of gene-editing technology. ZFN-mediated gene editing has yet to be reported in poultry. In contrast, TALEN-mediated gene targeting allowed the successful generation of ovalbumin (OVA) knockout chickens in 2014. Cultured primordial germ cells (PGCs) were transfected with plasmids encoding OVA-TALENs. This resulted in deletions in 33% of PGC cultures. The modified PGCs were transplanted into recipient embryos, producing chimeric roosters that, upon reaching sexual maturity, generated OVA heterozygous knockout chicks with a 10% efficiency [108]. In a similar approach, a 2017 study utilized TALENs combined with HDR to generate sterile hens. Cultured PGCs were transfected with TALEN-encoding plasmids, achieving an 8.1% editing efficiency. Heterozygous male PGCs were then transplanted into recipient embryos, and one of the resulting founder roosters successfully produced genetically modified offspring with a 6% efficiency [109]. These findings highlight the potential of TALENs for precise gene editing in poultry, though with relatively low efficiency.

On the contrary, in 2016, a study in chickens demonstrated successful germline gene editing through CRISPR-mediated homologous recombination in PGCs. An additional loxP site was inserted into the immunoglobulin heavy chain (IgH) locus via HDR, resulting in variable germline transmission rates across different PGC lines, with some reaching up to 90% [110].

Also, in 2016, Oishi et al. [111] achieved over 90% mutation efficiency in cultured chicken PGCs by targeting the OVA and ovomucoid genes using CRISPR/Cas9. Transplantation of CRISPR-modified ovomucoid PGCs into recipient embryos resulted in germline chimeric roosters, which transmitted the mutation to offspring at a rate of approximately 50%. Similarly, using CRISPR/Cas9, Koslová et al. [112] achieved a remarkably high efficiency of homologous recombination, with 88% of PGC clones successfully acquiring the precise deletion of the three nucleotides encoding the tryptophan residue at position 38 (W38) in both chNHE1 alleles.

Building on these studies and numerous other investigations [113,114], compelling evidence has demonstrated the superior efficiency of CRISPR/Cas9 over TALENs for gene editing in poultry. Beyond its higher mutation and transmission rates, CRISPR/Cas9 has surpassed TALENs as the preferred genome-editing tool due to its simplicity, speed, affordability, and greater target specificity. These advantages have driven its rapid adoption as the primary choice for precise genetic modifications across various organisms, including poultry.

CRISPR/Cas GE holds promise for addressing global food security challenges by enhancing production performances [98,115,116] and disease control measures [117–120]. It also offers opportunities to enhance animal welfare [121], create specific disease models [122], and develop poultry bioreactors [114].

**Figure 2:**
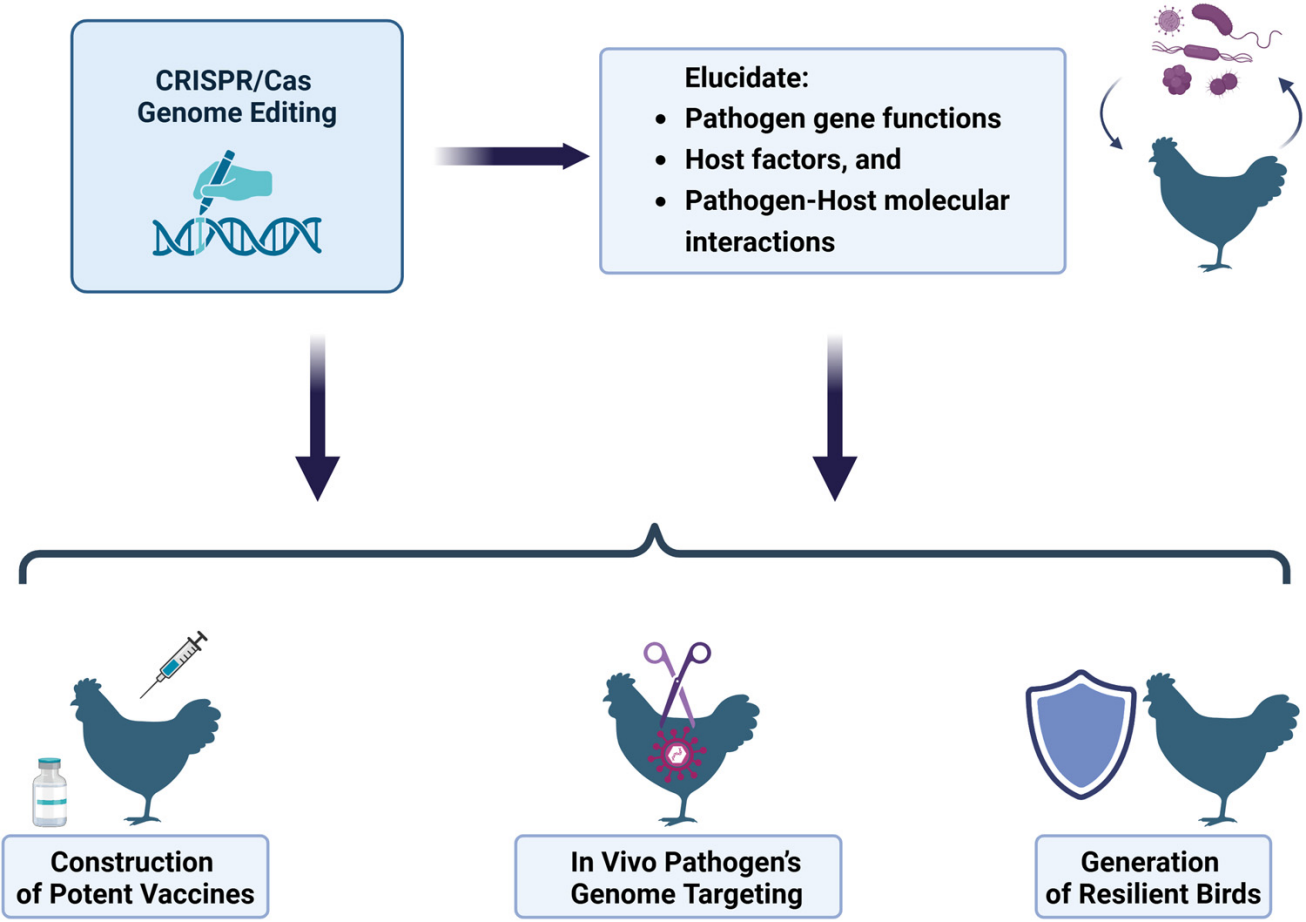
Leveraging CRISPR/Cas to control poultry infectious diseases.

The technique, which is capable of precisely targeting nearly any genomic location, has the potential to enhance traditional methods of disease prevention, control, or elimination. A primary benefit is the ability to develop disease-resilient poultry through genetic modifications in the host genome [123]. CRISPR/Cas genome editors can be applied to explore pathogen–host interactions, facilitate the engineering of vaccines, and *in vivo* pathogen targeting (Figure 2).

While the application of CRISPR/Cas in avian species is still emerging, notable progress has been achieved, primarily in two poultry species, namely chickens and quails, with chickens leading the way.

Beyond GE, certain CRISPR/Cas systems have found valuable applications in molecular diagnostics. Diagnostic tools based on CRISPR/Cas12a [124–128] and CRISPR/Cas13a [129–135] exhibit high efficacy, sensitivity, and speed in detecting poultry pathogens, with promising potential for point-of-care use.

By linking with transcriptional regulators or domains, catalytically inactive Cas9 (dCas9) can be employed to induce either the activation or repression of RNA transcription [136]. In this context, Williams et al. developed a novel genome and epigenome engineering toolkit enabling the manipulation of endogenous gene expression and enhancer activity in chicken embryos [137].

### 3.1 Use of CRISPR/Cas to elucidate pathogen’s gene functions, host factors, and pathogens–host molecular interactions

Advancements in genomics, particularly genome sequencing of both host and pathogens, empower researchers to identify candidate genes involved in infection and defense mechanisms.

CRISPR genome screening involves using CRISPR/Cas9 technology to systematically target and modify genes within a genome. The objective of this approach is to identify and study the function of specific genes by observing the resulting changes in cellular or organismal behavior [138,139]. This powerful tool offers new avenues for identifying relevant genes in hosts and pathogens during infection, as well as for investigating pathogen–host interactions [28,139–141]. These findings can be leveraged for identifying advantageous alleles for selective breeding within a poultry population, vaccine development [142] and to generate disease-resistant birds [143]. The successful editing process can be achieved with high efficiency by combining the suitable delivery strategy of Cas9 and sgRNA [144] with the right cells and CRISPR database tools.

Several studies have demonstrated the utility of CRISPR/Cas9 for functional gene analysis in poultry pathogens and host cells (Table 1).

**Table 1:** Studies using CRISPR/Cas to elucidate pathogen’s gene functions, host factors, and pathogen–host molecular interactions

Reference	Crispr/Cas9*-based gene edition	Key findings/study contribution
[145]	Deletion of the Meq and pp38 genes from the CVI988 vaccine strain of MDV	The first successful utilization of the CRISPR/Cas9 GE system for MDV-1 viral gene knockout
[146]	Deletion of the viral gene pp38 in MDV-transformed LCLs	The study contradicts prior reports and showcases that *pp38* gene is dispensable for the transformed state of MDV-transformed LCLs
[147]	Deletion of MDV-miR-M4 from the MDV-induced lymphoma-derived lymphoblastoid cell line MDCC-HP8	– MDV-miR-M4 is not essential for maintaining the transformed phenotype and continuous proliferation of LCLs – First study using the CRISPR/Cas9-based GE technology *in situ* to demonstrate that a critical virus-encoded miRNA is dispensable in maintaining the transformed phenotype of a virus-induced cancer cell line
[148]	Deletions of the Meq- or the mid-clustered miRNAs from vvMDV strain RB-1B virus	Establishing a new platform for mutagenesis of viral miRNAs encoded by the MDV-1
[149]	Deletion of the MDV-2 glycoprotein B (gB) in LCLs	Highlighting the potential of targeted GE as an antiviral strategy against pathogenic MDV-1 and other viruses affecting chickens
[150]	Deletion of MDV-1 pp38 creating a mutant (GX0101∆pp38) for the rapid screening and identification of pp38-specific monoclonal antibodies	– Identification of four specific antibodies against MDV-1’s pp38 protein – First demonstration of the use of CRISPR/Cas9-based GE technology for efficient screening and identification of mAbs against a specific viral protein
[151]	Generation of Meq-deleted MDV-1 viruses	– Presentation of a new efficient approach for the generation of specific mAbs against viral proteins – Identification of two mAb displaying high specificity against Meq protein
[152]	Remove the exon encoding the 33 amino acid insertion from chicken ANP32A (lacking in mammals) or knockout the entire protein in chicken cells	Influenza A viruses do not replicate in chicken cells that lack ANP32A
[153]	Deletion of the entire 99 nucleotide (nt) fifth exon (A99) of cANP32A	Chicken ANP32A only, not ANP32B and ANP32E, plays a pivotal role in supporting vPol activity of AIV
[154]	Deletion of candidate virulent ssaU gene encoding type III secretion system from *S.* Gallinarum genome	– Type III secretion system has a crucial role in mediating fowl typhoid’s virulence – First study that demonstrates a complete CRISPR/Cas9-based gene deletion from *S.* Gallinarum genome
[155]	Deletion of the SpvB gene from a large virulent plasmid of *S.* Gallinarum strain (SG18)	– SpvB deleted strain was found completely avirulent in chickens – First study to report a complete gene deletion from the *S.* Gallinarum virulent plasmid and its effect
[156]	– Disruption of *EtGRA9* gene in *E. tenella* genome – Disruption of each of the 33 members of the AP2 transcription factors family	– *EtGRA9* gene encodes a secreted protein whose cellular distribution varied during the parasite’s life cycle – Identification of 23 parasite factors that are essential for the development and survival in the host. First successful targeted gene disruption in *Eimeria* species
[157]	*: In this study, Cas12a was used instead of Cas9	Demonstration that FnCas12a could trigger GE in *E. tenella*
	Knocking-in a coding cassette for an enhanced yellow fluorescent protein (EYFP) and dihydrofolate reductase–thymidylate synthase gene (DHFR)	
[158]	Disruption of ETH2_0411800	ETH2_0411800 is non-essential for *E. tenella*’s growth and development
[159]	Disruption of the chicken TBK1 (*chTBK1*) gene in chicken embryonic fibroblast DF-1	chTBK1 was revealed to be indispensable in STING-mediated IFN-β activation in chicken cells
[160]	Recombination activating gene 1 (*RAG1*) knockout aiming the generation of an immunodeficient chicken model	Highlighting the pivotal role of *RAG1* in chicken immature B cell development, Ig gene conversion during embryonic stages, and demonstrates the dose-dependent regulatory role of *RAG1* during immune cell development
[161]	Deletion of chicken protein arginine methyltransferase 5 (*prmt5*) gene from DF-1 cell line	Uncovering a functional link of chPRMT5 in suppression of IFN-b production and interferon-stimulated gene expression
[162]	IRF7 knockout in DF-1 cells	IRF7 has a role in host antiviral response against the AIV in chickens

In 2018, Zhang et al. used CRISPR/Cas9 to knock out the *Meq* and *pp38* genes in serotype-1 Marek’s disease virus (MDV-1), revealing the potential of CRISPR for studying viral gene functions [145]. In 2019, the team extended this work to MDV-transformed cell lines, finding that pp38 deletion enhanced cell proliferation, suggesting it is non-essential for these cells’ transformation [146]. They also showed that MDV-miR-M4, a microRNA previously linked to tumor formation, is not necessary for maintaining the transformed phenotype [147]. By utilizing the CRISPR/Cas9 system with a double-guide RNA transfection/virus infection strategy, the team successfully established a new platform for mutagenesis of viral miRNAs encoded by the MDV-1 [148]. Further, in 2022, they successfully deleted MDV-2 glycoprotein B (gB) from MDV-transformed cells, showcasing GE’s potential as an antiviral strategy [149].

For MDV-specific antibody production, Teng et al. used CRISPR/Cas9 to create a pp38-deleted mutant for screening monoclonal antibodies (mAbs), identifying mAb 31G7 with high specificity [150]. Later, they developed Meq-specific mAbs using a hydrophilic polypeptide of Meq protein and hybridoma technology, proposing this technique for efficient mAb generation against viral proteins [151].

Avian influenza viruses (AIV) depend on specific host factors for replication, with variations in these factors affecting the virus’s ability to replicate in avian versus mammalian hosts [152]. In chickens, acidic nuclear phosphoprotein 32 family member A (ANP32A) has been identified as a critical host restriction factor for AIV. Park et al. used CRISPR/Cas9 to investigate the roles of ANP32 family members, finding that deleting a segment of chicken ANP32A, which includes an extra 33 amino acids not found in mammals, led to a significant reduction in viral polymerase activity. This result underscores the essential role of chicken ANP32A in supporting the AIV replication [153] and aligns with the observations of Long et al., who demonstrated that deleting ANP32B in chicken cells did not affect AIV polymerase activity. This indicates that ANP32A is the essential factor for AIV replication in chickens, while ANP32B and ANP32E are not involved in this process [152].

*Salmonella* Gallinarum, responsible for fowl typhoid, presents a significant economic threat to the poultry industry, particularly in developing countries. In 2022, researchers from the University of the Punjab, Pakistan, conducted gene deletion studies on *S.* Gallinarum [154,155]. Tahir et al. used CRISPR/Cas9 to delete the ssaU gene, which encodes the type III secretion system (TTSS), revealing its essential role in the pathogen’s virulence. The resulting mutant strain was non-virulent and unable to colonize poultry organs, suggesting potential use in live vaccine development [154]. Similarly, Basit et al. knocked out the SpvB gene, finding that infected chickens showed no signs of disease, supporting the use of CRISPR/Cas9 to develop attenuated vaccine strains [155].

Hu et al. applied CRISPR/Cas9 in *Eimeria tenella*, enabling single-gene and whole-gene family functional analysis. They identified 23 essential genes from the ApiAp2 transcription factor family, advancing the understanding of parasite development [156].

Cheng et al. optimized a transfection protocol utilizing FnCas12a protein for editing *E. tenella*, thereby enhancing opportunities for dissecting gene function and advancing the development of anticoccidial drugs and vaccines for *Eimeria* species [157]. CRISPR/Cas9-mediated disruption of ETH2_0411800 suggests that this gene is non-essential for *E. tenella*’s growth and development [158].

For poultry immune function studies, CRISPR/Cas9 facilitated gene knockouts in chicken DF-1 cells. One study demonstrated that the *TBK1* gene is vital for STING-mediated IFN-β activation in chicken cells [159]. Lee et al. used CRISPR/Cas9 to generate an immunodeficient chicken model by knocking out the *RAG1* gene [160].

Zeng et al. investigated the role of protein arginine methyltransferase 5 (PRMT5) in chicken cells. They generated a prmt5 gene-deficient DF-1 cell line using CRISPR/Cas9, which displayed increased IFN-b production compared to wild-type cells. This suggests a functional link of chPRMT5 in the suppression of IFN-b production and interferon-stimulated gene expression in chicken cells [161].

Additionally, research on IRF7-deficient chicken cells revealed increased viral replication of low pathogenic avian influenza virus, highlighting IRF7’s role in antiviral responses [162].

### 3.2 Construction of vaccines

Vaccines, alongside robust biosecurity measures, are central to disease prevention in the poultry industry. With the rise of emerging infectious diseases, enhancing vaccine strategies is critical. Recombinant multivalent vectored vaccines, which protect against multiple pathogens, are particularly valuable [163]. They can reduce selection pressure on field strains, streamline vaccination processes, lower production costs, and improve poultry welfare by reducing the need for multiple injections [164]. Vectored vaccines also elicit both cellular and humoral immune responses, and they facilitate DIVA (Differentiation Between Infected And Vaccinated Animals) strategies [165].

Over the past three decades, recombinant vectored vaccines, especially those using fowl pox virus and turkey herpesvirus (HVT), have become essential for controlling major viral diseases in poultry [166]. Advances in genetic engineering now enable the insertion of multiple foreign genes into vectors, allowing for broader disease protection [164]. CRISPR/Cas9 has further revolutionized this field by enabling the rapid development of multivalent vaccines that can simultaneously protect against several avian diseases [167,168] (Table 2).

**Table 2:** Studies using CRISPR/Cas to develop poultry vaccines

Vector	Antigen encoded by the inserted expression cassette	Disease(s) targeted by the candidate vaccine^1^	Reference
HVT	AIV H7N9 HA	AI (H7HA subtypes)	[176]
	IBDV VP2	IBD	[177]
	ILTV gD-gI and the AIV H9N2 HA (insertion into the previously developed HVT-IBDV VP2 viral genome)	– ILT – AI (H9N2 subtype) – IBD	[168]
	F (Fusion) gene of NDV	ND	[178]
	OmpH gene from *P. multocida*	Fowl cholera	[180]
	IBDV (G2d strain) VP2 gene	IBD (especially due to the IBDV G2d strain)	[181]
	HA of AIV H9N2 (Y280 strain)	AI (H9N2 Y280 strain)	[182]
	– IBDV (G2d strain) VP2 gene – HA of AIV H9N2 (Y280 strain)	IBDV (G2d) + AIV (H9N2/Y280)	[183]
	Insertion of mCherry cassette aiming the identification of new potential sites for the insertion of foreign genes.	A novel intergenic site HVT-005/006 was identified.	[175]^2^
	HA of the H9N2 was inserted in this new site	Confirmation of the suitability of HVT-005/006 site for inserting foreign genes	
DEV	HPAIV H5N1 HA	– AI (H5Nx subtypes) – DTMUV infection	[187]
	Pre-membrane proteins (PrM) and envelope glycoprotein (E) of DTMUV		
	H5N8-HA	Avian influenza (H5Nx subtypes)	[186]
	GFP was used as a tag and removed later by Cre-Lox		
	Outer membrane protein H (ompH)	Fowl cholera	[167,189]
FAdV-4	Fusion protein of RFP and FAdV-4 Fiber-1	Hepatitis hydropericardium syndrome (HHS)	[191]
	Fusion protein of EGFP and FAdV-4 Fiber-2		[192]
	Fiber-2 without N-terminal 7–40aa		[193]
	EGFP replacing Fiber-2		[194]
	Fiber of FAdV-8b	HHS and IBH	[195]
	HA of the H9N2 AIV	– HHS – AI (H9N2 subtype)	[196]
*Eimeria acervulina*	– 12 copies of extracellular domain of AIV H9N2 M2 (M2e) protein – Tags: EYFP, RFP	Coccidiosis (*E. acervulina*)	[197]
ILTV	NDV Fusion (F) protein (in addition to the deletion of vector’s thymidine kinase [TK] and unique short 4 [US4] genes)	ILT and ND	[198]

^1^In addition to Marek’s disease for chicken when the vector is HVT and in addition to Duck Viral Enteritis in susceptible species when the vector is DEV. ^2^In this study, the objective was to identify novel insertion sites rather than the construction of a vaccine targeting a specific disease. GFP: green fluorescence protein, RFP: red fluorescence protein, EGFP: enhanced green fluorescence protein, EYFP: enhanced yellow fluorescent protein.

#### 3.2.1 Hvt-based candidate vaccines constructed using CRISPR/Cas9-mediated gene-editing technique

HVT has been used for decades as a vaccine against Marek’s disease (MD) [169], a highly contagious poultry disease characterized by the development of T-cell lymphomas and nerve enlargement. Vaccination, coupled with sanitation and selective breeding, is the primary control strategy for MD. HVT, with its large double-stranded DNA genome [170], can accommodate foreign genes encoding immunogenic proteins. Its non-pathogenic nature and ability to induce long-lasting immunity made it an early choice for expressing foreign antigens, enabling HVT to simultaneously provide immunity against MD and other viral diseases [171–173] avoiding interference between individual vaccines [174].

CRISPR/Cas9 has further advanced HVT vaccine development by streamlining the creation of multivalent vaccines, increasing the potential for HVT to serve as a versatile vector in poultry vaccination strategies.

Zai et al. identified a new insertion site within HVT, enabling stable expression of foreign genes, demonstrated by the successful integration of H9N2 hemagglutinin [175]. They recommended screening the entire HVT genome to discover additional sites for foreign gene insertion.

Chang et al. developed a bivalent HVT vaccine by inserting the H7N9 hemagglutinin gene into a specific intergenic region of HVT [176]. Similarly, Tang et al. [168] created a triple-insert recombinant vaccine by incorporating the infectious laryngotracheitis virus (ILTV) gD-gI gene and AIV H9N2 hemagglutinin into an HVT strain already expressing the VP2 protein from infectious bursal disease virus (IBDV) [177]. This innovative vaccine offers protection against three major avian diseases alongside MD.

The rHVT-F vaccine, expressing the fusion (F) protein of genotype XII Newcastle disease virus (NDV), provided full protection in chickens assessed five days post-challenge [178]. In a follow-up study, the F gene of genotype XII NDV was inserted into two different sites within the HVT genome. A single dose of the resulting vaccines provided sustained protection for at least 52 weeks post-vaccination [179].

Fowl cholera, caused by the highly transmissible bacterium *Pasteurella multocida*, is a significant avian ailment with global implications. Apinda et al. engineered rHVT-OmpH, carrying an outer membrane protein gene from *P. multocida*. This recombinant vaccine induced strong immunity and protected ducks from the pathogen, showing HVT’s potential for non-chicken hosts [180].

To counter the IBDV (G2d) variant, researchers developed rHVT-VP2, achieving full protection against this challenging strain [181]. More recently, the rHVT/Y280 vaccine was engineered by inserting the hemagglutinin (*HA*) gene of H9N2/Y280 into the HVT genome, conferring protection against H9N2/Y280 [182]. Subsequently, the *VP2* gene of IBDV (G2d) was added to this recombinant virus, creating the dual-insert rHVT-VP2-HA vaccine for broader immunization coverage [183].

#### 3.2.2 Duck enteritis virus (DEV)-based candidate vaccines constructed using CRISPR/Cas9-mediated gene-editing technique

DEV, a highly fatal alpha-herpesvirus affecting ducks, geese, and swans [184], has been repurposed as a vector for recombinant multivalent vaccines due to its large genome and restricted host range [185–188]. Zou et al. developed a novel recombinant DEV (rDEV) encoding genes for HPAIV H5N1 and duck tembusu virus (DTMUV), creating a trivalent vaccine (C-KCE-HA/PrM-E). Ducks vaccinated with this candidate showed strong immune responses and were protected against all three pathogens [187].

Using an NHEJ-CRISPR/Cas9 and Cre-Lox system, another rDEV was engineered to express influenza antigens. This system allowed for green fluorescence protein (GFP) tagging, followed by its removal [186]. Apinda et al. applied a similar method to develop rDEV vaccines expressing the *P. multocida* OmpH gene at two genomic sites [167,189]. These recombinant viruses matched the growth characteristics of wild-type DEV [189] and successfully protected ducklings against both DEV and *P. multocida*, without inducing any clinical symptoms or vaccine-related pathology [167].

#### 3.2.3 Fowl adenovirus-based candidate vaccines constructed using CRISPR/Cas9-mediated gene-editing technique

Hepatitis-hydropericardium syndrome (HHS), caused by highly virulent Fowl Adenovirus (FAdV) serotypes, especially FAdV-4, poses a significant economic threat to the poultry industry [190]. Researchers from Hangzhou University in China have developed several live attenuated recombinant FAdV-4 vaccine candidates using CRISPR/Cas9 technology, publishing their findings in six articles between 2021 and 2023. These candidates include FAdV4-RFP_F1, which expresses the fusion protein of red fluorescence protein (RFP) and Fiber-1 [191]; and FA4-EGFP, which expresses the enhanced green fluorescence protein EGFP-Fiber-2 fusion protein [192]. Another candidate, FAV-4_Del, involves a deletion within Fiber-2 [193], while FAdV4-EGFP-rF2 replaces Fiber-2 entirely with EGFP [194]. These vaccines showed significant attenuation and provided complete protection against FAdV-4 in chicken trials [191–194]. The efficacy of FAdV4-EGFP-rF2 as a recombinant vaccine candidate, despite the knockout of the entire fiber-2 gene, illustrates its dispensability for both FAdV-4 virus replication and effective protection [194]. To broaden protection, the team created FA4-F8b, a recombinant virus expressing FAdV-8b fiber, aimed at preventing both HHS and inclusion body hepatitis (IBH). FA4-F8b was inactivated due to its high pathogenicity in 2-week-old SPF chicks, but it still provided effective protection against both FAdV-4 and FAdV-8b after inactivation [195].

In their latest study, the researchers used a double-fluorescence system to further modify FadV-4 [192], producing FAdV4-HA(H9), which expresses the HA gene from H9N2 AIV. This candidate vaccine was attenuated, induced early immune responses, and reduced H9N2 replication in chickens [196].

#### 3.2.4 Candidate recombinant vaccines based on other vectors

##### 3.2.4.1 Eimeria acervulina

Zhang et al. achieved stable transfection of *E. acervulina*, with confirmed expression of the AIV H_9_N_2_ M2 (M2e) protein in the cytoplasm of sporozoites. The fecundity of the modified parasite (EaM2e) matched that of the wild type [197]. Subsequent investigations are required to determine whether EaM2e can serve as a live vaccine vector.

##### 3.2.4.2 Infectious laryngotracheitis virus

Atasoy et al. used a CRISPR/Cas9 system combined with the Cre-Lox system to simultaneously delete virulence factors and insert foreign genes into the ILTV genome. They successfully removed the thymidine kinase (TK) and unique short 4 (*US4*) genes while adding the NDV fusion (F) gene. This method did not impair ILTV replication or F protein expression, providing a promising tool for creating attenuated and multivalent vaccine vectors [198].

### 3.3 *In vivo* pathogen’s genome targeting

CRISPR/Cas could be used to precisely cut pathogen’s genomes in a targeted sequence offering the potential to prevent or treat infections [199].

Li et al. efficiently edited the long terminal repeats of reticuloendotheliosis virus (REV) using CRISPR/Cas9, resulting in the inhibition of viral protein expression and the disruption of the proviral genome in chicken cells. Furthermore, they successfully delivered the CRISPR/Cas9 system into REV-infected chickens using an attenuated MDV vaccine strain as a vector. This led to a reduction in REV viral load and alleviation of associated symptoms [200]. This marks the first instance of using herpesvirus-delivered CRISPR/Cas9 to confer resistance against avian retroviruses in chickens, providing a novel strategy against viral infections.

Challagula et al. explore the use of CRISPR/Cas13a to selectively disrupt RNA in chicken fibroblast DF1 cells, particularly focusing on its potential as an antiviral strategy against the influenza A virus (IAV). The team designed multiple CRISPR RNAs (crRNAs) against IAV genes, demonstrating reduced viral titers in cells transfected with these crRNAs. The study suggests that Cas13a’s precision and lack of off-target effects make it a promising tool for functional studies and antiviral strategies in chickens, with the potential to combat HPAIV strains [201].

The same year, Challagula et al. reported the development of transgenic chickens expressing Cas9 and guide RNAs (gRNAs) targeting the *ICP4* gene of MDV. These chickens showed significantly reduced MDV replication when challenged with the virus. The designed gRNAs specifically interfered with MDV replication in transgenic chicken cells but not with HVT, suggesting that CRISPR/Cas9 can be used as an antiviral approach to control MDV infection in chickens without impeding the use of HVT as a vector for recombinant vaccines [202].

Mohsin et al. applied CRISPR/Cas9 to disrupt *E. tenella* genes, showing a remarkable reduction in lesion and oocyst scores, supporting its use against parasitic infections [203].

Liu et al. assessed the effectiveness of using MDV as a delivery system for the CRISPR/Cas9 gene-editing tool to target and disrupt the reverse-transcribed products of the avian leukosis virus subgroup J (ALV-J) RNA genome during its infection cycle *in vitro* and *in vivo*. They showed that the engineered MDV, expressing ALV-J-targeting CRISPR/Cas9, successfully resisted ALV-J challenges in host cells. This outcome demonstrates the CRISPR/Cas9 system’s effectiveness as a treatment against ALV-J infection and suggests the potential of MDV as an efficient delivery system for CRISPR/Cas9 in chickens [204].

Recent advancements in CRISPR/Cas9 GE highlight its transformative potential in targeting integrated viral genomes. A recent study demonstrated the safety and efficacy of CRISPR/Cas9 for *in vivo* editing of proviral DNA in simian immunodeficiency virus (SIV)-infected rhesus macaques. Targeting multiple regions of the SIV genome, the study achieved functional biodistribution to SIV reservoirs without off-target effects or abnormal pathology. Notably, macaques receiving higher doses showed improved lymphocyte counts, underscoring the therapeutic promise of this approach [205]. Like ALV-J, SIV is a retrovirus, and the ability to excise its DNA from host genomes provides a compelling proof of concept for using CRISPR to tackle integrated viral genomes. This breakthrough opens possibilities for eradicating persistent retroviral pathogens in poultry, such as Marek’s disease virus or lymphoid leukosis virus. By demonstrating the feasibility of excising integrated viral DNA *in vivo*, these findings set a precedent for developing CRISPR-based therapies for persistent poultry pathogens.

Several species of Mycoplasma cause substantial economic losses in livestock. The challenge in studying and addressing these bacteria lies in the lack of efficient recombination and genome engineering tools, hindering the production of mutant strains for identifying virulence factors and developing improved vaccine strains for many Mycoplasma species. Ipoutcha et al. developed an effective CRISPR-derived genetic tool for introducing targeted mutations in three major pathogenic species among them, the avian species *Mycoplasma gallisepticum* (Mgal). The team employed an inducible dCas9-cytidine deaminase system to disrupt several major virulence factors in these pathogens. Individual mutants of potential virulence genes were isolated [206].

### 3.4 Generation of disease-resilient poultry

In the context of production animals, disease resilience refers to an animal’s ability to maintain productive performance in the face of infection [207,208], and it encompasses two key components: disease resistance and disease tolerance. Disease resistance is defined as the individual’s ability to inhibit or limit pathogen replication within the host [209], while disease tolerance refers to the infected host’s capacity to reduce the impact of infection on health and performance, enabling it to sustain high levels of health or production despite a given pathogen load within the host [120].

Traditional breeding methods, which involve techniques such as cross-breeding and selective breeding, have been relied upon to enhance desirable traits in livestock, including disease resilience. However, they are limited by the natural genetic variation within populations and can be time-consuming [210,211]. While conventional breeding programs have led to gradual improvements, they may encounter challenges in obtaining disease resilience if many genes are involved [120].

The use of precise gene editing (PGE) in poultry is considered a transformative technology, offering the potential to revolutionize the breeding of desired traits in livestock [118]. PGE allows for the swift incorporation of new or existing beneficial mutations within a species or closely related ones that do not typically interbreed into elite breeding animals while avoiding the introduction of unwanted traits typical of traditional selective breeding [212].

In certain instances, although similar genetic improvements could theoretically be achieved through traditional breeding methods, GE expedites the process by bypassing the need for multiple generations of selective crossings or the identification of rare animals carrying desired genetic variants [119]. While GE is not intended to replace traditional breeding, it complements it by providing breeders with increased genetic variation to select from [213,214].

With recent advances in precision genome targeting, the generation of genetically modified poultry is now more attainable than ever [215]. The introduction of resilience alleles into a poultry population could be obtained by editing host factors crucial for pathogens’ entry or replication. Viruses, for example, rely on specific host cell receptor molecules to enter target cells [216]. Through the precise targeting and removal or modification of these receptors, CRISPR/Cas9-mediated GE has the potential to effectively hinder viral infections [217]. However, regulatory frameworks and public acceptance are still evolving, and further research is needed to fully realize the potential of GE.

The development of porcine reproductive and respiratory syndrome (PRRS)-resistant pigs using CRISPR/Cas9 GE highlights the transformative potential of this technology in combating economically devastating livestock diseases. By precisely targeting the porcine *CD163* gene, which encodes a receptor essential for viral entry, researchers achieved complete resistance to the PRRS virus [218–221]. This groundbreaking solution addresses one of the most economically damaging illnesses for swine producers [222,223]. A scaled gene-editing program successfully introduced this resistance trait into four genetically diverse and elite porcine lines, ensuring its relevance and applicability for commercial breeding populations [224]. This achievement exemplifies how precise GE can target critical host factors to confer disease resistance and serves as a model for similar innovations in poultry.

#### 3.4.1 *In vitro* testing for resistance to pathogens

Preliminary *in vitro* trials using specific cell cultures are essential for evaluating the efficacy and safety of genetic modifications aimed at developing disease-resistant poultry.

Lymphoid leukosis is caused by avian leukosis viruses (ALVs), which are categorized into subgroups A, B, C, D, and J [225]. Control measures focus on eradicating the virus from breeding flocks [20]. This strategy has substantially reduced the frequency of the disease in commercial flocks. Given the susceptibility of all studied chicken lines to ALV infection [226], there is considerable interest in developing resilient chicken lines.

To induce resistance to infections by ALV subgroups B [227], J [228], and A [229], Lee et al. utilized CRISPR/Cas9-based GE to modify viral receptor genes in DF-1 chicken fibroblasts. The tumor virus locus B (*tvb*) gene, encoding the TVB receptor, which is essential for ALV subgroup B entry into host cells, was efficiently modified, conferring resistance to ALV subgroup B [227]. For ALV-J, they altered the ALV-J receptor: the chicken Na+/H+ exchange 1, (chNHE1) by targeting the tryptophan residue at position 38 (Trp38) [228] previously characterized as involved in viral attachment and entry [230]. The targeted mutation resulted in a complete resistance to viral infection. Similarly, disruption of exon 2 within the *tva* gene in DF-1 fibroblasts conferred resistance to ALV subgroup A. Using a sequential approach, they modified all three receptor genes to block ALV subgroups A, B, and J, demonstrating the potential for generating cells resistant to various viral pathogens by targeting distinct receptors for cellular entry [229].

Koslová et al. similarly used CRISPR/Cas9 to introduce frame-shifting mutations in chicken cell line DF-1 at the *tvc*, *tva*, and *tvj* loci, conferring resistance to ALV subgroups C, A, and J [231]. These findings paved the way for creating ALV-resistant chickens using CRISPR/Cas9 [112].

The limited pathogenicity of AIV in waterfowl, such as ducks, is attributed to the presence of the retinoic acid-inducible gene I (RIG-I) [232], in contrast to chickens, which lack this gene and exhibit severe disease when infected with HPAIV. To confer resistance to AIV in chickens, RIG-I was successfully knocked into chicken DF-1 cells, establishing a RIG-I-dependent immune response without overexpression of RIG-I or disruption of host genes [233].

Another study involved replacing the C-terminal domain (CTD) of chicken melanoma differentiation-associated protein 5 (cMDA5) with that of RIG-I. The engineered *cMDA5* gene was then expressed in cMDA5 knockout DF-1 cells. This modification resulted in an enhanced interferon-mediated immune response and a notable reduction in the titer of IAV [234].

#### 3.4.2 Advances in methods for *in vivo* gene editing utilizing the CRISPR/Cas9 system in poultry

##### 3.4.2.1 Cultured PGCs-mediated gene editing in poultry

PGCs, the embryonic precursors of sperm and egg cells, can be isolated from various stages of embryonic development [235–237] and cultured while maintaining their germline competency [238]. When cultured PGCs are introduced into the bloodstream of recipient embryos, they migrate to the gonads and integrate into the germline, resulting in the creation of a germline chimera [239,240]. Cultured PGCs allowed the generation of the first knockout chickens in 2013 [241]. The *in vitro* genetic editing of chicken PGCs using CRISPR/Cas9 system has become a standard practice in many chicken research laboratories, opening up numerous potential applications for genetically edited chickens [110,113,242].

When injected into a host chick embryo, edited PGCs integrate into the host embryo’s gonads alongside the native PGCs, diminishing the likelihood of offspring deriving from the donor PGCs in subsequent mating [243]. To address this, methods to reduce or eliminate native PGCs have been explored, but they often fail to eradicate all germ cells and pose significant toxicity risks to the host embryo. Recently, Ballantyne et al. developed a surrogate host chicken line allowing for conditional ablation of both male and female germlines. By using CRISPR/Cas9-mediated HDR to target the DAZL gene, which is exclusively expressed in the germ cell lineage, they induced apoptosis in the host’s germ cells upon activation of caspase-9 protein by a chemical compound. This enables efficient colonization of the host’s gonads by edited PGCs. Direct mating of these surrogates facilitates the production of pure-breed homozygous edited offspring, reducing generation time and increasing the number of homozygous genome-edited offspring [244].

While cultured PGCs serve as effective tools for GE in poultry, there are limitations to this method. Notably, only chicken PGCs have been reliably cultured long-term *in vitro*. This limitation makes it difficult to isolate and amplify genome-edited PGCs in other species. Moreover, PGC-based techniques are time-intensive, involving the selection of edited PGCs, microinjection, and raising G0 germline chimeras until sexual maturity to obtain edited offspring.

##### 3.4.2.2 *In vivo* transfection of PGCs

The direct injection of genome engineering tools into the circulatory system of the developing embryo just before the PGCs migrate to the gonads enabled the transformation of circulating PGCs and the generation of transgenic chickens [215,245]. This method could be applied to species that lack the long-term PGC culture method.

##### 3.4.2.3 Sperm Transfection–Assisted Gene Editing (STAGE)

STAGE entails directly transfecting spermatozoa with Cas9 mRNA and sgRNA. Using these modified sperm for adult hen insemination allows for the direct production of edited progeny. Although GE has been achieved successfully in chicken embryos using STAGE, the efficiency of producing edited offspring remains relatively low, indicating the need for further enhancements [246].

##### 3.4.2.4 Viral infection

The direct injection of the adenoviral CRISPR/Cas9 vector into the avian blastoderms was successfully applied to generate genome-edited quail [101] and was later applied to generate edited chicken and duck [247].

##### 3.4.2.5 Other methods

Intracytoplasmic sperm injection (ICSI)-mediated GE technology is a rapid method to generate targeted gene knockout in poultry [248,249]. Nevertheless, implementing this technique demands substantial technical expertise and effort, along with specialized equipment. The current hatching success rate remains modest, indicating the need for additional research.

#### 3.4.3 Disease-resilient chickens developed using CRISPR/Cas9 systems

ALV-J replication relies on the functional cellular receptor chNHE1, where a crucial amino acid for virus entry is the tryptophan residue number 38 (W38) located in its extracellular segment [228,230]. Building on these findings, Koslová et al. deleted W38 in chicken PGCs. Edited PGCs underwent an orthotopic transplantation and successfully developed, with significantly elevated efficacy, an inbred chicken line CB resistant to ALV-J infection with no observable side effects in edited birds [112]. Also, by precisely deleting W38, Hellmich et al. successfully conferred ALV-J resistance in a commercial egg-type chicken line [250]. Edited birds challenged by a highly pathogenic ALV-J displayed no pathological clinical signs or lesions [251].

Although earlier studies reported promising outcomes, recent *in vitro* and *in vivo* findings by Matoušková et al. reveal that minor modifications to the ALV-J receptor NHE1, specifically the deletion of a single amino acid (W38), initially block ALV-J effectively but may ultimately be circumvented by viral adaptations in the envelope protein. These findings suggest that more substantial receptor alterations may be required to achieve durable resistance [252].

In order to generate a chicken line resistant to ALV A and K, Koslová et al. edited chicken PGCS (CPGCs) by introducing a frame-shifting deletion into the chicken *tva* gene coding the Tva cell surface protein serving as the entry receptor for ALV A and K. Edited cells are then transplanted into the testes of sterilized recipient roosters. The resulting *tva*−/− chickens demonstrated complete resistance to ALV A and K in both *in vitro* and *in vivo* assessments, contrasting with their susceptible *tva*+/+ and *tva*+/− siblings. The *tva* knockout chickens exhibited a specific disorder in cobalamin/vitamin B12 metabolism, aligning with the recognized role of Tva as a receptor for cobalamin [253]. To address this concern, the authors suggest a more targeted modification of Tva by changing a specific amino acid residue crucial for virus binding, such as the C40W substitution found in the *tva* allele.

Globally, the poultry production industry faces significant challenges from AIV H_9_N_2_ infections and HPAI outbreaks, resulting in heavy economic losses. Moreover, several AIV serotypes are zoonotic with the risk of the emergence of strains with pandemic potential [254].

Controlling AI through poultry vaccination faces challenges due to rapid and continuous antigenic drift in field viruses and global limitations in vaccine production and supply [255]. GE has emerged as a promising solution to develop AIV-resistant poultry [256].

In chicken cells, the AI viral RNA polymerase depends on chicken ANP32A proteins for replication [257], while ANP32B is inactive [153,258]. Researchers used CRISPR/Cas9 to induce N129I and D130N substitutions into the ANP32A gene [259] to impede AIV infection and transmission in chickens. The residues were altered in CPGCs, and gene-edited chickens were subsequently derived from these modified cells [143]. Gene-edited chickens showed resistance to AIV (H9N2-UDL) infection through natural transmission routes when exposed to infected birds without displaying health issues. However, very high inoculation dose led to breakthrough infections with various amino acid substitutions detected in the viral polymerase genes, enabling the enzyme to utilize the edited ANP32A protein and suboptimal ANP32 family members [143]. This unintended outcome underscores the importance of a robust GE strategy and subsequent evaluation, including challenges with various AI phenotypes at non-physiological exposure levels to eliminate the possibility of adapted viruses eruption.

Ultimately, editing all three members of the ANP32 family in chicken cells, resulting in no virus polymerase activity, illustrated a proof of principle for combining multiple edits in host genes to confer sterile resistance. However, the potential deleterious effects on animal health highlight the need for careful consideration [143]. For a successful strategy against AI, the authors suggest multiple edits targeting the proviral potential of ANP32A, B, and E in order to eliminate the risk of escape mutants [143].

This study marks a significant milestone in genetic research as GE has successfully generated viable chickens partially resistant to influenza virus A infection for the first time.

## 4 Conclusion and perspectives

Poultry, serving as a significant protein source, is facing various challenges, including infectious diseases, resulting in considerable economic losses and public health concerns.

Over the past decade, CRISPR/Cas9-mediated GE technology has undergone rapid advancement. Due to its precision, efficiency, versatility, and simplicity, the system has revolutionized genetic modification, offering the potential to enhance the prevention and control of poultry infectious diseases.

Through targeted modifications at specific loci, this technology has significantly advanced our understanding of host–pathogen interactions. The insights gleaned have contributed to the swift creation of novel candidate poultry vaccines and have facilitated the development of disease-resistant birds. Moreover, this technology enables *in vivo* targeting of pathogens, marking a pivotal stride forward in bolstering infectious disease prevention and control efforts.

Leveraging CRISPR/Cas9-based strategies, innovative multivalent vectored vaccines have been engineered, offering the potential for simultaneous protection against up to four major poultry diseases [168]. The efficacy of the developed candidate vaccines has been remarkable, coupled with a notable absence of adverse reactions. This suggests a promising trajectory toward the commercial availability of CRISPR-engineered poultry vaccines in the market.

Recent studies have showcased the efficacy of CRISPR/Cas technology in targeting a multitude of poultry pathogens. CRISPR/Cas13a has been effectively utilized to disrupt AIV RNA in chicken cells, demonstrating its potential as an antiviral strategy. Additionally, CRISPR/Cas9 has been successfully employed to target specific DNA loci in the genomes of viruses, bacteria, and parasites, resulting in the inhibition of their replication within the host.

Producing permanent disease resilience, which can be passed down through generations, is a key objective in poultry production. It allows for the reduction of culling, vaccination costs, and surveillance programs. By integrating GE with poultry breeding programs, it becomes possible to develop poultry lines with enhanced disease resilience.

Targeted deletion of the W38 residue in the ALV-J receptor NHE1 confers initial resistance in chickens [112,250]; however, recent findings have demonstrated the eruption of resistant viral strains, suggesting a need for more extensive receptor modifications [252].

Target edition to the *ANP32A* gene resulted in resistance to AIV H9N2-UDL without adverse effects on health or productivity [143]. However, challenges remain, as evidenced by breakthrough infections observed at higher viral doses, highlighting the importance of robust editing strategies and continued evaluation to mitigate potential risks of viral adaptation.

Despite the tremendous potential of CRISPR/Cas systems, several challenges need to be addressed. Off-target effects, delivery methods, ethical concerns, public acceptance, and regulatory discrepancies across countries remain key barriers to widespread application.

A major concern is whether gene-edited poultry will be accepted by the public, as past experiences with genetically modified organisms (GMOs) suggest that public perception plays a crucial role in the adoption of new biotechnologies [260]. GMOs produced using earlier technologies faced strong opposition, often driven by concerns over food safety, environmental impact, and corporate control over food production [261]. Given this historical context, genome-edited poultry, despite not containing foreign transgenes like traditional GMOs, may still encounter similar challenges in public acceptance [262]. Misconceptions about gene editing and distrust in regulatory institutions could contribute to resistance unless proactive efforts are made to communicate the distinctions between CRISPR-based GE and conventional genetic modification. One of the central factors influencing public acceptance is the purpose of GE. Reports suggest that people are more likely to support gene editing when it is used to enhance animal health and welfare, such as preventing infectious diseases, rather than for productivity gains that primarily benefit producers [263]. Public attitudes toward genome-edited poultry are also shaped by the perceived risks associated with off-target effects and genetic stability. Ethical concerns related to animal welfare emphasize the importance of minimizing unintended genetic modifications, as unforeseen mutations could lead to physiological or behavioral changes with negative implications for livestock well-being [264].

Ensuring that GE aligns with ethical considerations, including minimizing animal suffering and maintaining genetic diversity, could help build broader acceptance [262].

Transparent communication, rigorous safety assessments, and strong governance frameworks are necessary to foster trust in genome-editing applications. Engaging the public in discussions on the ethical and societal implications of this technology – while ensuring that regulatory policies align with broader societal values – will be crucial in determining the long-term acceptance of gene-edited poultry. Ultimately, fostering trust in the institutions responsible for gene editing and demonstrating tangible benefits for both animals and consumers will be key to integrating CRISPR-based innovations into poultry production responsibly and sustainably.

Regulatory hurdles remain a significant challenge in the commercialization of genome-edited poultry, including those produced using CRISPR/Cas9. Current frameworks in many countries fail to distinguish genome-edited animals from transgenic organisms, subjecting them to lengthy and costly approval processes. For example, in the United States, the FDA regulates genetically modified animals as “New Animal Drugs,” leading to approval timelines exceeding 15 years, as seen with genetically engineered pigs [265] and fish [266]. Similarly, in the European Union, rigid policies do not differentiate between transgenic and genome-edited animals, further complicating approvals [267]. In contrast, countries like Brazil [268] and Argentina [269] have streamlined their regulations, recognizing genome-edited animals (without foreign DNA insertions) as equivalent to traditionally bred animals, avoiding unnecessary regulatory barriers. China, a leading country in CRISPR/Cas9 research [270,271], actively supports GE advancements but still regulates genome-edited animals under GMO laws, with no commercial approvals to date [272]. However, regulatory landscapes are evolving, reflecting a global trend toward balancing innovation and regulation [273]. The European Parliament’s recent vote on New Genomic Techniques (NGTs), which exempts gene-edited plants deemed indistinguishable from conventionally bred ones from GMO legislation, signals a shift toward more adaptable policies [274].

Amid current challenges, the optimism surrounding CRISPR/Cas systems remains well-founded. With the ongoing advancements in next-generation sequencing and artificial intelligence, CRISPR/Cas applications are expected to broaden, encompassing additional poultry species such as turkeys, geese, ducks, and guinea fowl. Researchers are hopeful that continued advancements and collaborative efforts will address current limitations, ultimately enabling the full potential of GE technologies. This optimistic trajectory points toward a future where CRISPR/Cas can be harnessed to achieve sustainable and resilient poultry production on a global scale, transforming the industry and providing significant benefits for disease resistance, environmental sustainability, and animal welfare.
